# Breast carcinoma in situ: An observational study of tumor subtype, treatment and outcomes

**DOI:** 10.18632/oncotarget.13785

**Published:** 2016-12-02

**Authors:** Qi Wu, Juanjuan Li, Si Sun, Shan Zhu, Chuang Chen, Juan Wu, Qian Liu, Wen Wei, Shengrong Sun

**Affiliations:** ^1^ Department of Breast and Thyroid Surgery, Renmin Hospital of Wuhan University, Wuhan, Hubei, P. R. China; ^2^ Department of Clinical Laboratory, Renmin Hospital of Wuhan University, Wuhan, Hubei, P. R. China; ^3^ Department of Pathology, Renmin Hospital of Wuhan University, Wuhan, Hubei, P. R. China

**Keywords:** breast carcinoma in situ, tumor subtype, treatment, outcomes

## Abstract

**Background & Aims:**

To evaluate the clinical presentation, treatment and outcome of patients with breast carcinoma in situ (BCIS) with special emphasis on the role of the tumor subtype and local treatment in these patients.

**Methods:**

Using data obtained by the Surveillance, Epidemiology, and End Results (SEER) program from 2010-2013, a retrospective, population-based cohort study was conducted to investigate tumor subtype-specific differences in various characteristics, overall survival (OS) and breast cancer-specific mortality (BCSM).

**Results:**

In all, 6867 patients with BCIS were eligible during the 2010-2013 study period. Compared with the hormone receptor (HoR)+/HER- subgroup, patients with triple negative (TN) breast cancer were more likely to have tumors that were higher in grade and larger in size; they were also more likely to have tumors with ductal and comedo histology and were less likely to have tumors with cribriform and papillary histology (each P < 0.05). During the follow-up period, patients with TN breast cancer had an OS of 97.0% compared with 98.6 % in the HoR+/HER- subgroup (P < 0.05). Furthermore, the BCSM rate was 1.0% for the TN group compared with 0.1% for the HoR+/HER- subgroup (P < 0.05). Multivariate analysis revealed that patients with TN MBC had a poorer OS and BCSM (P <0.05). Multivariate analysis of OS with respect to the local treatment history showed that patients who received breast-conserving surgery (BCS) combined with radiotherapy (R) were more likely to have an improved OS (P < 0.05). Moreover, the results demonstrated that patients who underwent SLNB were more likely to have a lower BCSM (P < 0.05).

**Conclusions:**

The results demonstrate that BCIS appears to alter the prognosis associated with the TN subtype. Meanwhile, BCS plus R was a preferable option and resulted in survival rates that were better than those achieved with mastectomy; thus, SLNB should be considered as an appropriate assessment of axillary staging in patients with BCIS.

## INTRODUCTION

The term breast carcinoma in situ (BCIS) encompasses lesions that contain abnormal epithelial cells that are completely confined within breast lobules and/or ducts without invasion beyond the basement membrane. BCIS includes a variety of pathological types. The 2 major types of breast carcinoma in situ are ductal carcinoma in situ (DCIS) and lobular carcinoma in situ (LCIS). The incidence of BCIS increased rapidly after the introduction of mammography as a population screening tool and has subsequently increased at a slower rate [1–4].

In the majority of patients, BCIS is primarily viewed as an indicator of an increased risk for invasive breast cancer. Moreover, several studies have revealed that BCIS lesions tend to be small in size, grade II or III, and widely positive for estrogen receptor (ER) and progesterone receptor (PR), but HER2 testing is not a routine part of the pathologic evaluation [5, 6]. However, studies have also suggested that high nuclear grade DCIS lesions are often negative for ER and that they overexpress HER2. Furthermore, this subtype may be associated with reduced survival, and thus targeting HER2 is a potential treatment strategy for HER2-overexpressing DCIS. Additionally, a new Van Nuys Prognostic Index (VNPI) is used as an independent predictor of local recurrence; this new index has a new formula that accounts for tumor size, margin width, pathologic classification, and age [7].

Traditional treatment has been mastectomy, whereas breast-conserving surgery (BCS) is a feasible surgical option for select patients. In addition to BCS, the effect of sentinel lymph node biopsy (SLNB) in BCIS remains unclear. Based on the current standards, SLNB has been recommended as a less invasive method compared with axillary lymph node dissection (ALND) for the staging of patients with early invasive ductal carcinoma. We speculate that SLNB might be approved as an effective method to detect axillary lymph nodes (ALNs) in patients with BCIS. The benefit of radiotherapy in terms of a significantly reduced risk of local recurrence (LR) in those who receive BCS has been demonstrated by several large randomized controlled trials [8, 9]. However, the idea that radiotherapy should be avoided in selected low-risk cases remains uncertain.

The clinical characteristics of BCIS and the optimal approaches to treatment are topics of uncertainty and concern for both patients and clinicians. Therefore, this article will evaluate the clinical presentation, treatment and outcomes of patients with BCIS, with special stress on the role of breast cancer subtype, BCS and SLNB.

## RESULTS

### Clinical and tumor characteristics

In all, 6867 patients with BCIS were eligible during the 2010-2013 study period. We excluded 54,190 patients whose medical records did not contain information on breast cancer subtype and 241 patients whose survival times were classified as unknown in the analysis. Information was available for 4324 patients with BCIS in the HoR+/HER- subgroup, 1409 patients in the HoR+/HER+ subgroup, 795 patients in the HoR-/HER+ subgroup and 429 patients in the triple-negative (TN) subgroup, who were all included in this study.

Differences in patient demographics, cancer characteristics, treatments, and outcomes among the subgroups are summarized in Table 1. Compared with the HoR+/HER- subgroup, patients with TN breast cancer were more likely to have tumors that were higher in grade and larger and were more likely to have tumors with ductal and comedo histology; these patients were less likely to have tumors with cribriform and papillary histology (each P < 0.05). Patients within the HoR-/HER+ subgroup had tumors that were, in general, higher in grade and larger in size compared with tumors of patients in the HoR+/HER- subgroup. Furthermore, patients in the HoR-/HER+ subgroup were the most likely to have tumors with comedo and papillary histology. With respect to treatment options, patients within the four subgroups tended to receive breast-conserving surgery (BCS) and sentinel lymph node biopsy (SLNB).

**Table 1:** Patient characteristics within subgroups

Variables	HoR+/HER-N=4324 (%)	HoR+/HER+N=1409(%)	HoR-/HER+N= 705(%)	TNN= 429(%)	P value^*^
**Follow-up(months)**	21.61±13.93	21.67±14.19	21.59±13.57	23.31±13.91	
**Age at diagnosis, y**					P < 0.001
** <35**	28(0.6)	14(1.0)	5(0.7)	0(0.0)	
** 35-49**	883(20.4)	335(23.8)	128(18.2)	68(15.9)	
** 50-64**	1747(40.4)	642(45.6)	322(45.7)	173(40.3)	
** ≥65**	1666(38.5)	418(29.7)	350(35.5)	188(43.8)	
**Sex**					0.039
** Female**	4298(99.4)	1402(99.5)	705(100)	428(99.6)	
** Male**	26(0.6)	7(0.5)	0(0.0)	1 (0.4)	
**Race**					P < 0.001
** white**	3307(76.5)	1110 (78.8)	575(81.6)	333(77.6)	
** Black**	62(14.5)	170(12.1)	62(8.8)	60(14.0)	
** Other**	351(8.1)	115(8.2)	62(8.8)	34(7.9)	
** Unknown**	39(0.9)	14(1.0)	6(0.9)	2(0.5)	
**CHSDA Region**					0.197
** East**	2225(51.5)	702(49.8)	330(46.8)	205(47.8)	
** Northern Plains**	541(12.5)	190(13.5)	99(14.0)	54(12.6)	
** Pacific Coast**	1452(33.6)	475(33.7)	256(36.3)	156(36.4)	
** Southwest**	106(2.5)	42(3.0)	19(2.7)	13(3.0)	
** Alaska**	0(0)	0(0)	1(0.1)	1(0.2)	
**Grade**					P < 0.001
** Well **	772(17.9)	71(5.0)	8(1.1)	15(3.5)	
** Moderately **	1798(41.6)	371(26.3)	70(9.9)	82(19.1)	
** Poorly **	1014(23.5)	707(50.2)	488(69.2)	257(59.9)	
** Undifferentiated**	102(2.4)	80(5.7)	60(8.5)	26(6.1)	
** Unknown**	638(14.8)	180(12.8)	79(11.2)	49(11.4)	
**Histology**					P < 0.001
** Ductal**	1694(39.2)	621(44.1)	285(40.4)	204(47.6)	
** Lobular**	148(3.4)	22(1.6)	6(0.9)	9(2.1)	
** Ductal and Lobular**	107(2.5)	25(1.8)	5(0.7)	8(1.9)	
** Ductal with other types**	1339(31.0)	436(30.9)	190(27.0)	101(23.5)	
**Paget disease**	2(0)	11(0.8)	19(2.7)	3(0.7)	
** Comedo**	288(6.7)	179(12.7)	165(23.4)	76(17.7)	
** Cribriform**	448(10.4)	65(4.6)	20(2.8)	12(2.8)	
** Papillary**	239(5.5)	39(2.8)	8(18.1)	8(1.9)	
** Intracystic**	49(1.1)	5(0.4)	1(1.1)	0(0)	
** Others**	10(0.2)	6(0.4)	6(0.9)	8(1.9)	
**Tumor size(mm)**					P < 0.001
** ≤ 10**	2700(62.4)	788(55.9)	334(47.4)	214(49.9)	
** 10-20**	876(20.3)	302(21.4)	187(26.5)	116(27.0)	
** 20-50**	587(13.6)	240(17.0)	147(20.9)	81(18.9)	
** > 50**	161(3.7)	79(5.6)	37(5.2)	18(4.2)	
**Laterality**					0.505
** Left**	2264(52.4)	723(51.3)	383(54.3)	201(46.9)	
** Right**	2059(47.6)	686(48.7)	322(45.7)	228(53.1)	
**Radiotherapy**					P < 0.001
** No**	2218(51.3)	667(48.0)	360(51.1)	204(47.6)	
** Yes**	2001(46.3)	690(49.0)	321(45.5)	216(50.3)	
** Unknown**	105(2.4)	42(3.0)	24(3.4)	9(2.1)	
**Surgery**					P < 0.001
** Mastectomy**	1283(29.7)	493(35.0)	274(38.9)	154(35.9)	
** BCS**	2923(67.6)	876(62.2)	407(57.7)	266(62.0)	
** Unknown**	118(2.7)	40(2.8)	24(3.4)	9(2.1)	
**LN surgery **					P < 0.001
** SLNB**	4082(94.4)	1325(94.0)	649(92.1)	393(91.6)	
** ALND**	198(4.6)	76(5.4)	44(6.2)	31(7.5)	
** Unknown**	44(1.0)	8(0.6)	12(1.7)	4(0.9)	
**Status**					P < 0.001
** Alive**	4263(98.6)	1387(98.4)	695(98.6)	416(97.0)	
** Dead**	61(1.4)	22(1.6)	10(1.4)	13(3.0)	
** Breast cancer**	6(0.1)	6(0.4)	3(0.3)	4(1.0)	
** Other**	55(1.3)	16(1.2)	7(1.1)	9(2.0)	

* P values calculated by Pearson Chi squared testing; Bold if statistically significant, p< 0.05

*y: years, mm:* *millimeter, y: years, BCS: breast-conserving surgery, HoR:* *hormone receptor, TN: triple negative, LN: lymph node, SLNB:* *sentinel lymph node biopsy, ALND: axillary lymph node dissection.*

### Survival analysis

**Figure 1:**
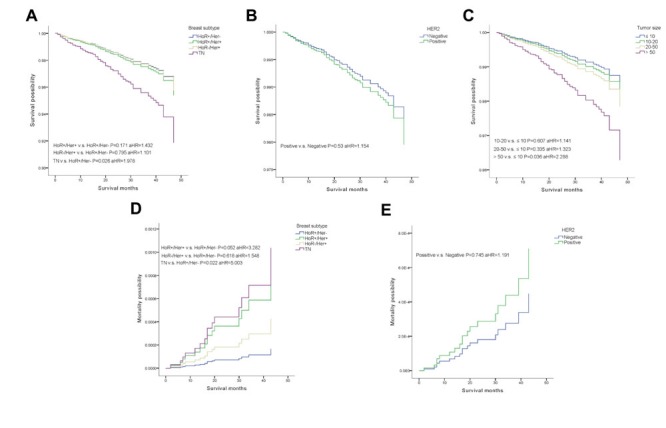
Weighted Kaplan-Meier curves of overall survival(OS) and breast-cancer-specific mortality(BCSM) **A.** OS is based on tumor subtype. **B.** OS is based on HER2 status. **C.** OS is based on tumor size. **D.** BCSM is based on tumor subtype. E. BCSM is based on HER2 status.

A weighted Kaplan-Meier analysis was used to determine overall survival (OS) and breast cancer-specific mortality (BCSM), which were based on breast cancer subtype and HER2 status, of the patients in the subgroups. Survival curves for the subgroups were generated (Figure 1). At the median follow-up of 22 months, patients with TN breast cancer had an OS of 97.0% compared with patients in the HoR+/HER- subgroup who had an OS of 98.6 % (P < 0.05). In addition, the BCSM rate was 1.0% for the TN group compared with 0.1% for the HoR+/HER- subgroup (P < 0.05).

We used a multivariate analysis based on the weighted Kaplan-Meier results. All the prognostic factors that predicted OS and BCSM were analyzed in a multivariate analysis (Table 2). In the multivariate analysis, patients with TN breast cancer were more likely to have a poorer OS and a higher BCSM compared with patients in the HoR+/HER- subgroup, as shown in Figure 1A, 1D (OS, P = 0.026, aHR = 1.978; BCSM, P = 0.022, aHR = 5.003). Unexpectedly, these findings showed a decreased OS only in the subgroup of patients whose tumor size was > 50 (Figure 1C, OS, P = 0.036, aHR = 2.288; BCSM, P = 0.54, aHR = 1.688). Furthermore, HER2 status was not associated with OS or BCSM (Figure 1B, 1E, OS, P = 0.53, aHR = 1.154; BCSM, P = 0.745, aHR = 1.191).

**Table 2:** Cox proportional hazards regression model analysis of overall survival (OS) and breast cancer-specific mortality (BCSM)

Variables	OS		BCSM	
	aHR (95% CI)	P-value	aHR (95% CI)	P-value
**Age at diagnosis, y**				
** <35**	Reference		Reference	
** 35-49**	501.3(0.0,2.393)	0.171	980.6(0.0,6.91E96)	0.95
** 50-64**	1240.3(0.0,2.275)	0.795	1103.7(0.0,7.75E96)	0.949
** ≥65**	4086.3(0.0,3.605)	0.061	3567.1(0.02,2.5E97)	0.941
**Sex**				
** Female**	Reference		Reference	
** Male**	0.0(0.0,6.4E48)	0.898	0.56(0.0,5.9E135)	0.959
**Race**				
** white**	Reference		Reference	
** Black**	0.608(0.321,1.153)	0.128	0.56(0.12,2.617)	0.461
**Grade**				
** Well **	Reference		Reference	
** Moderately **	0.903(0.493,1.653)	0.74	0.697(0.123,3.996)	0.684
** Poorly **	0.662(0.338,1.293)	0.228	1.008(0.188,5.408)	0.993
** Undifferentiated**	1.024(0.353,2, 969)	0.965	1.176(0.091,15.175)	0.901
**Histology**				
** Ductal**	Reference		Reference	
** Lobular**	0.785(0.234,2.629)	0.695	0.001(0.0,9,7E42)	0.125
** Ductal and Lobular**	0.371(0.051,2.713)	0.329	0.0(0.0,2,7E61)	0.563
** Ductal with other types**	0.909(0.554,1.489)	0.704	1.164(0.369,3.67)	0.563
** Paget disease**	1.807(0.412,7.926)	0.443	0.0 (0.0,4.9 E105)	0.125
** Comedo**	1.341(0.717,2.508)	0.359	1.226(0.309,4.86)	0.563
** Cribriform**	0.547(0.214,1.397)	0.207	0.001(0.0,9.5E27)	0.563
** Papillary**	1.158(0.511,2.625)	0.725	1.282(0.152,10.802)	0.125
** Intracystic**	0.689(0.091,5.238)	0.719	0.0 (0.0,8.1E116)	0.563
** Others**	0.0(0.0, 3.9E56)	0.91	0.0(0.0,4.2E164)	0.563
**Tumor size(mm)**				
** ≤ 10**	Reference		Reference	
** 10-20**	1.141(0.69,1.889)	0.607	0.235(0.029,1.892)	0.174
** 20-50**	1.323(0.749,2.337)	0.335	0.981(0.243,3.961)	0.978
** > 50**	2.288(1.055,4.963)	0.036	1.688(0.317,9.001)	0.54
**ER**				
** Negative**	Reference		Reference	
** Positive**	1.302(0.694,2.441)	0.411	1.481(0.41,5.353)	0.549
**PR**				
** Negative**	Reference		Reference	
** Positive**	0.577(0.332,1.002)	0.051	0.314(0.089,1.107)	0.072
**HER2**				
** Negative**	Reference		Reference	
** Positive**	1.154(0.739,1.802)	0.53	1.191(0.415,3.418)	0.745
**Subtype**				
** HoR+/HER-**	Reference		Reference	
** HoR+/HER+**	1.432(0.857,2.393)	0.171	3.282(0.989,10.888)	0.052
** HoR-/HER+**	1.101(0.533,2.275)	0.795	1.548(0.279,8.598)	0.618
** TN**	1.978(1.087,3.60)	0.026	5.003(1.263,19.821)	0.022
**Radiotherapy**				
** No**	Reference		Reference	
** Yes**	0.303(0.18,0.51)	P < 0.001	0.466(0.104,2.098)	0.32
**Treatment**				
** Mastectomy**	Reference		Reference	
** BCS**	1.707(1.005,2.899)	0.048	0.979(0.226,4.242)	0.977
** BCS+R**	0.499(0.286,0.869)	0.014	0.295(0.073,1.198)	0.088
**LN surgery **				
** SLNB**	Reference		Reference	
** ALND**	1.677(0.828,3.399)	0.151	3.902(1.104,13.788)	0.035

* P values calculated by Log-rank testing; Bold if statistically significant, p< 0.05

*BCS: breast-conserving surgery, R:* *Radiotherapy, HoR:* *hormone receptor, TN: triple negative, LN: lymph node, SLNB:* *sentinel lymph node biopsy, ALND: axillary lymph node dissection.*

*aHR: adjusted hazard ratio (adjusted for age at diagnosis, race, grade,* *histology, tumor size, laterality, ER, PR, HER2, subtype, radiotherapy, treatment and LN surgery).*

### Effect of surgical treatment on survival outcomes

**Figure 2:**
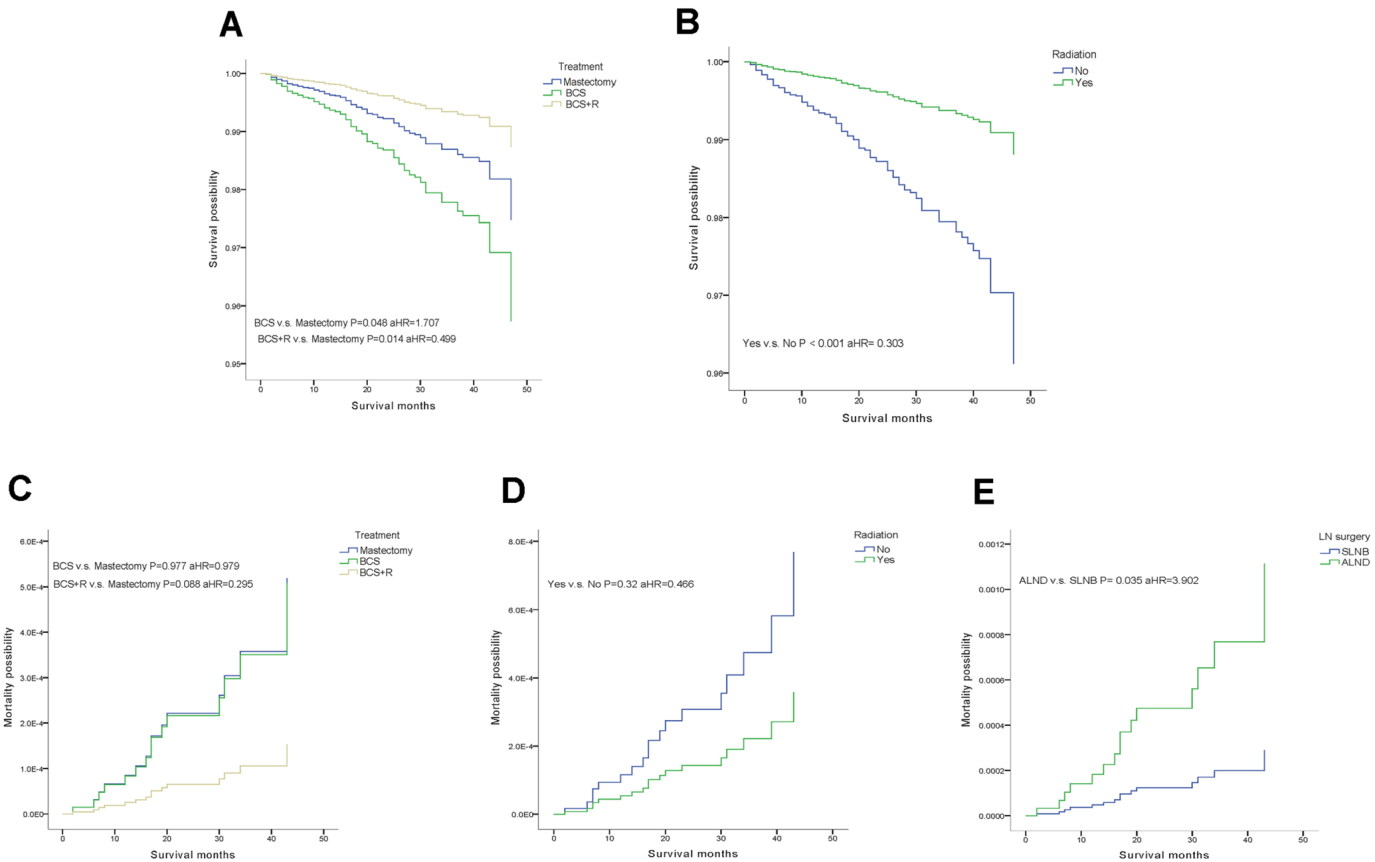
Weighted Kaplan-Meier curves of overall survival(OS) and breast-cancer-specific mortality(BCSM) in subgroup analysis. A. OS is based on treatment. B. OS is based on radiotherapy. C. BCSM is based on treatment. D. BCSM is based on radiotherapy. E. BCSM is based on lymph node (LN) surgery.

We characterized three subgroups, based on different local treatments, as follows: mastectomy, BCS only and BCS with radiotherapy (BCS+R) (Table 2, Figure 2). During the follow-up period, a multivariate analysis of OS based on local treatment history showed that patients who underwent BCS combined with radiotherapy (R) were more likely to experience an improved OS compared with those who underwent mastectomy (P = 0.014, aHR = 0.499). Additionally, patients who underwent BCS only tended to have a lower OS (Figure 2A, P = 0.048, aHR = 1.707). We also used the analysis to examine the option of radiotherapy, which showed that patients who underwent radiotherapy had a higher OS that did not affect the BCSM (Figure 2B, 2D, OS, P < 0.001, aHR = 0.303; BCSM, P = 0.32, aHR = 0.466). However, the results demonstrated a significant difference within the LN surgery only group in terms of BCSM, and patients who underwent SLNB were more likely to have a lower BCSM (Figure 2E, P = 0.035, aHR = 3.902).

## DISCUSSION

In this large population-based cohort of cases diagnosed with BCIS, we found a poorer survival in patients with the TN subtype after adjustment for other factors. In addition, our analysis of tumor size demonstrated a tumor size > 50 mm was more likely to augment the OS of patients with BCIS. In our series, a dramatic difference was observed within the surgical treatment and radiotherapy subgroups with respect to OS, where patients who underwent SLNB were more likely to have a lower BCSM.

In previous studies, nuclear grade, tumor size and age were all important factors that could be used to predict local recurrence in patients with BCIS [10, 11]. However, our research revealed that nuclear grade and age were not prognostic factors for OS or BCSM, and our findings showed a decreased OS only in patients with a tumor size > 50. In the current study, patients in the HoR-/HER+ subgroup had tumors that were generally higher in grade and larger in size compared with tumors of patients in the HoR+/HER- subgroup. The correlation between HER2 and tumor behavior has been previously described. One study revealed that HER2 was overexpressed in 24/31 (77%) patients with DCIS who experienced local relapse [12]. In contrast, another study showed that HER2 overexpression may not be the key factor in the progression of DCIS to invasive carcinoma and that HER2 gene amplification is inversely related to invasive progression in patients with DCIS [13, 14]. However, the precise incidence of HER2 overexpression in many cases of DCIS is unclear. A retrospective analysis was performed and showed that HER2 was overexpressed in 61% of cases of DCIS [15]. In contrast, Roses et al. [16] reported 106 patients with DCIS and noted HER2 overexpression in only 37% of cases. They also described an association between HER2 overexpression and the detection of invasive foci in surgical specimens. However, we analyzed common prognostic factors as well as HER2 status, and the results showed that HER2 status was not correlated with survival after adjustment for other prognostic factors. This conclusion remains to be confirmed by a future prospective trial.

Triple-negative (TN) BCIS was seldom diagnosed; this may be because tumors of the TN subtype progress quickly. Therefore, our study reported that patients with TN breast cancer tended to have tumors that were higher in grade and larger in size; their tumors were also more likely to have ductal and comedo histology, and their prognosis was more likely to be poor. In contrast to a previous study, our study reported that tumors of the TN subtype may progress much faster than other tumor types [14]. Some reports have suggested that TN BCIS may be a potential precursor to TN invasive cancer [17, 18], and a more frequent and rapid progression from BCIS to invasive cancer was found to be related to the comedo histologic subtype of BCIS [19, 20]. Consequently, some unrecognized mechanisms or features that promote the progression of the TN subtype should be further studied.

The surgical treatment of BCIS is controversial. Although mastectomy has been demonstrated to be curative for approximately all patients with BCIS [21, 22], mastectomy represented significant overtreatment for the majority of cases detected by the current methods. When BCS was elected rather than mastectomy, radiotherapy statistically decreased local recurrence rates compared with BCS alone [23]; nevertheless, radiotherapy may also represent overtreatment for low-risk patients. Several studies revealed that local excision only was an appropriate surgery for patients with low-risk BCIS [7, 24]. However, BCS plus radiation for moderately or high-risk patients was the consensus. A retrospective study showed that the overall survival was similar for all three subgroups (BCS alone, BSC+R and mastectomy), but the addition of radiation to BCS decreased the LR from 43% to 7% [25]. In addition, several retrospective studies[26–30] reported the survival outcome of patients with DCIS who were managed by BCS with or without radiotherapy and mastectomy. As expected, BCS alone resulted in consistently higher rates of LR (range, 8%-34%) compared with patients treated by BCS+R (range, 0%-17%). Our results also demonstrated that patients who underwent BCS+R were more likely to exhibit an improved OS, and patients who underwent BCS alone tended to have a lower OS compared with those in the mastectomy subgroup. Therefore, the recommendation of BCS combined with radiotherapy was the preferable alternative for patients with BCIS. As in the cited studies, axillary metastases were observed in approximately 1-2% of BCIS cases [31]. Due to the associated increased risk of coexisting microinvasion, the need for axillary staging becomes more relevant[32, 33]. SLNB, which was used as an approach in the common population of patients with BCIS, appeared to be feasible [34–36]. In the present study, patients who underwent SLNB exhibit a similar survival with those who received ALND regardless of the surgical method. Likewise, our results reported that patients treated by SLNB had a similar OS and better BCSM in comparison with those who received ALND. Future studies are warranted to determine the potential benefits of SLNB in patients with BCIS.

When the molecular profiles of BCIS are considered, some studies confirmed that BCIS demonstrated high expression of estrogen receptor (ER), which was associated with low-grade lesions, but these tumors were positive for c-erbB-2, Ki-67 and p53, the expression of which is associated with high-grade lesions [31, 37, 38]. A recent case-control study suggested that BCIS cases that were triple-positive for p16, COX-2 and Ki67 had a significantly higher rate of progression to invasive breast cancer than those that were negative for these biomarkers (8-year risks for subsequent invasive cancer were 19.6% and 4.1%, respectively) [39]. Simultaneously, several studies confirmed that HoR negativity, high S-phase fraction, abnormal DNA ploidy, p53 overexpression and HER2 overexpression were associated with more aggressive tumor behavior in BCIS [40–43]. Gene-expression profiling is likely to enhance our understanding of BCIS behavior and its relationship to invasive breast cancer. Findings of several studies [40, 44, 45] recorded differential expression patterns and identified new facets of the earliest stage of breast-cancer progression. More molecular and genetic studies that predict local recurrence and progression to invasive breast cancer independent of standard prognostic markers are required, and the difference in survival must continue to be monitored.

The main limitations of this study were the heterogeneous population and its retrospective setting. The information on systemic therapy and margin control was insufficient, and the follow-up was limited. As a result, HER2 targeted therapy and novel adjuvant hormone therapy remained in use for the management of BCIS to significantly improve the survival. Additionally, we had no specific information on the type of axillary surgery, and thus we substituted the number of lymph nodes removed.

Despite the limitations, our study demonstrates that BCIS appears to alter the prognosis associated with the TN subtype. Moreover, BCS plus R was the preferable option and resulted in survival rates better than those achieved with mastectomy. SLNB should be considered as an appropriate assessment of axillary staging in patients with BCIS.. However, the surgical treatment plan must be chosen for its strength in aiding the clinical and imaging assessment. Further studies are needed to minimize variation in modes of treatment and to establish a standardized management approach.

## MATERIALS AND METHODS

### Data source and study design

We collected data obtained between 2010 and 2013 from the National Cancer Institute's Surveillance, Epidemiology, and End Results (SEER) program. SEER began to collect information on HER2 status in 2010. Therefore, we used that year as the starting point. We used the International Classification of Diseases for Oncology, 3rd edition (ICD-O-3) histopathology codes to extract all cases with BCIS (codes 8201, 8230, 8500 through 8507, 8523). ICD-O-3 codes were also used to categorize BCIS cases by subtype (DCIS, not otherwise specified [8500, 8523], comedo carcinoma [8501], papillary [8503], micropapillary [8507], cribriform [8201], solid [8230], other [8502, 8504–8506] and LCIS [8520, 8524]) based on a scheme that has been previously described [46]. We selected cases with known hormone receptor (HoR) status and HER2 status. Patients who underwent surgery, those with an unknown type of breast cancer and patients who were diagnosed at autopsy were excluded.

Demographic variables included age at diagnosis ( < 35, 35-49, 50-64, >65 years) and race (white, black, other). Cancer characteristics were classified according to grade (well, moderately, poorly, undifferentiated, unknown), tumor size (≤ 10, 10-20, 20-50, > 50 mm), laterality (right, left, others, unknown), HoR status and HER2 status (positive, negative, borderline, unknown). Treatment characteristics included receipt of radiation therapy (no, yes, unknown). The subtypes were characterized according to the breast subtype variable as either HoR+/HER2-, HoR+/HER2+, HoR-/HER2+ or triple-negative (TN). Patients were categorized according to whether they underwent BCS (surgery of primary site variable values of 20-24) or mastectomy (surgery of primary site variable values of 30-80). Since the type of axillary surgery was not reported within the SEER database, the patients who had 1-5 lymph nodes removed were regarded as the sentinel lymph node biopsy (SLNB) group and those with > 5 lymph nodes that were removed were regarded as the axillary lymph node dissection (ALND) group, as in previous studies [47].

The two primary outcomes in our study were overall survival (OS) and breast cancer-specific mortality (BCSM). Vital stats were reported as either “alive” or “dead” in the SEER dataset. The survival time (in months) was calculated for each patient using the “Completed Months of Follow-up” given in the SEER database. The overall survival (OS) was determined by patients who were alive at the end of the study period or who were alive at their last follow-up. Breast cancer-specific mortality (BCSM) was determined by a comparison of patients whose cause of death was due to breast cancer with patients who were alive at the end of the study period, those who had died from other causes, or who were alive at their last follow-up. Cases without survival times were classified as unknown and were removed from the study.

### Statistical analysis

Patient demographics and cancer- and treatment-related characteristics were compared among the subgroups using Chi square or Fisher's exact tests. Survival outcomes on OS and BCSM were estimated using the weighted Kaplan-Meier method, and variables were compared among the subgroups using the log-rank test. Univariate and multivariate Cox proportional hazard regressions were used to obtain hazard ratios (HRs) and their respective 95% confidence intervals to show the strength of the estimated relative risk; these approaches were applied to model the relationship between potential covariates and either OS or BCSM. All statistical analyses and all charts of survival probabilities were performed with SPSS 19.0 (IBM Corporation, Armonk, NY, USA). A two-sided P value < 0.05 was considered statistically significant.
